# Cataract Surgery Visual Outcomes and Associated Risk Factors in Secondary Level Eye Care Centers of L V Prasad Eye Institute, India

**DOI:** 10.1371/journal.pone.0144853

**Published:** 2016-01-07

**Authors:** Sumathi Matta, Jiwon Park, Ghanshyam Palamaner Subash Shantha, Rohit C. Khanna, Gullapalli N. Rao

**Affiliations:** 1 Andhra Pradesh Right to Sight Society, Hyderabad, India; 2 Massachusetts Institute of Technology, Cambridge, United States of America; 3 Department of Internal Medicine, University of Iowa Hospitals and Clinics, Iowa city, IA, United States of America; 4 Allen Foster Research Centre for Community Eye Health, Gullapalli Pratibha Rao International Centre for Advancement of Rural Eye care, L V Prasad Eye Institute, Hyderabad, India; 5 Brien Holden Eye Research Centre, L.V. Prasad Eye Institute, Banjara Hills, Hyderabad, India; University of Illinois at Chicago, UNITED STATES

## Abstract

**Purpose:**

To evaluate cataract surgery visual outcomes and associated risk factors in rural secondary level eye care centers of L V Prasad Eye Institute (LVPEI), India.

**Methods:**

The Eye Health pyramid of LVPEI has a network of rural secondary care centres (SCs) and attached vision centres (VCs) that provide high quality comprehensive eye care with permanent infrastructure to the most disadvantaged sections of society. The most common procedure performed at SCs is cataract surgery. We audited the outcome of a random sample of 2,049 cataract surgeries done from October 2009-March 2010 at eight rural SCs. All patients received a comprehensive ophthalmic examination, both before and after surgery. The World Health Organization recommended cataract surgical record was used for data entry. Visual outcomes were measured at discharge, 1–3 weeks and 4–11 weeks follow up visits. Poor outcome was defined as best corrected visual acuity <6/18.

**Results:**

Mean age was 61.8 years (SD: 8.9 years) and 1,133 (55.3%) surgeries were performed on female patients. Pre-existing ocular co-morbidity was present in 165 patients (8.1%). The most common procedure was small incision cataract surgery (SICS) with intraocular lens (IOL) implantation (91.8%). Intraoperative complications were seen in 29 eyes (1.4%). At the 4–11 weeks follow-up visit, based on presenting visual acuity (PVA), 61.8% had a good outcome and based on best-corrected visual acuity (BCVA), 91.7% had a good outcome. Based on PVA and BCVA, those with less than 6/60 were only 2.9% and 1.6% respectively. Using multivariable analysis, poor visual outcomes were significantly higher in patients aged ≥70 (OR 4.63; 95% CI 1.61, 13.30), in females (OR 1.58; 95% CI 1.04, 2.41), those with preoperative comorbidities (odds ratio 4.68; 95% CI 2.90, 7.57), with intraoperative complications (OR 8.01; 95% CI 2.91, 22.04), eyes that underwent no IOL or anterior chamber-IOL (OR 12.63; 95% CI 2.65, 60.25) and those undergoing extracapsular cataract extraction (OR 9.39; 95% CI 1.18, 74.78).

**Conclusions:**

This study demonstrates that quality cataract surgeries can be achieved at rural SCs. The concept of the LVPEI SCs can be applied to other developing countries, allowing rural patients to attain better vision through cataract surgery. Despite improvements in quality of cataract surgery, gender discrimination in terms of outcome continues to be an issue and needs further investigation.

## Introduction

Globally, cataract is the major cause of blindness accounting for 51% of total blindness [1] and there are regional variations in it [2,3] with America having the lowest prevalence and the highest being in South-East Asia [2,3]. Apart from the presence of cataract, outcomes of cataract surgery are also an issue in many locations in the world. Unlike hospital based studies [4–7], numerous population based studies have shown wide variability in terms of post-operative visual outcomes, both within a country [8,9] as well as between countries [10–14]. There is also urban-rural differences seen [8]. This variability in outcomes could be due to the quality of surgery or the surgical facility, surgeons’ skills, post-operative use of spectacles or co-existing ocular co-morbidities. These differences can also be due to the fact that population-based studies include all cases, some of which might have been operated on many years ago. It is also likely that there is a reporting bias in hospital-based studies, with hospitals having good outcomes reporting on a regular basis, whereas others do not. However, this outcome only represents the communities’ interpretations and expectations. Hence, there is a need to follow standardize cataract surgical protocols, surgical skills as well as post-operative follow-up care. Apart from this, there is also need to routinely monitor the outcomes of cataract surgery.

L V Prasad Eye Institute (LVPEI) has developed pyramidal model of eye care with a Centre of Excellence (CoE) at the top and community-based Vision Health Guardians (VHG) at the bottom of the pyramid [15]. In between are Vision Centres (VCs), Secondary Centres (SCs) and Tertiary Centres (TCs). Cataract surgery as a procedure is done in the CoE, TCs and SCs. Throughout the eye health pyramid, there is an uniform protocol followed and skills imparted to perform high quality cataract surgery. This study assessed the outcomes of cataract surgeries done in eight secondary level service centers of LVPEI as well as analyzing the risk factors for poor outcomes.

## Material and Methods

The Ethics Committee of the L V Prasad Eye Institute, Hyderabad, India, approved this study and it was conducted in accordance with the tenets of the Declaration of Helsinki. As it was retrospective analysis of medical records and identity of each patient was kept anonymous, consent were not needed.

In this retrospective study, cataract surgery records in eight secondary care service centers of L V Prasad Eye Institute were randomly selected and examined. The selected secondary care service centers represented five districts across the Indian states Andhra Pradesh and Telengana- three from Prakasam, two from Adilabad, and one each from Mahabubnagar, Nellore, and Chittoor districts. Data of patients having cataract surgeries were collected randomly from the Medical Records Department (MRD) at each secondary center from October 2009 to March 2010. Only age-related cataract surgery records of patients aged ≥40 years were included in the study. Surgery records involving traumatic cataract, combined procedures and secondary intraocular lens implantation (IOL) were excluded. However, if the first surgery happened in the given period (October 2009-March 2010) and if the patient was left aphakic and had secondary IOL implantation during the same period (October 2009-March 2010), data related to primary surgery was only included. This was done to avoid double counting the same patient as well as the existing World Health Organization (WHO) recommended software for data entry allows for patients undergoing cataract surgery only and not for aphakic patients.

All patients examined at the secondary centers for cataract surgery received a comprehensive ophthalmic examination. The standard pre-operative examination included a detailed history, measurements of presenting visual acuity (PVA) and best corrected visual acuity (BCVA) with Snellen charts, intraocular pressure measurement with applanation tonometry, detailed slit lamp examinations including a dilated examination to assess the lens status as well as ocular comorbidities likely to affect the outcome.

Standard Operating Procedures (SOP) and definitions specified in the World Health Organization (WHO) Manual for Monitoring Cataract Surgical Outcomes (MCSO) were followed [16]. Data was retrospectively entered by each centre in the WHO recommended cataract surgical record (CSR) form and analyzed [16]. Double entry was done in database and number of in- built checks are present in software package to avoid data entry errors and regular check was done by randomly selecting 20 records to check whether all data have been entered correctly or not.

Ocular comorbidities were grouped as corneal scar, old iritis, retinal disease, glaucoma and others. The surgeons at the secondary centers, depending on the cataract grading, his or her experience with the procedure and the pupil status, determined the surgical technique. Procedures performed were manual small incision cataract surgery (MSICS), phacoemulsification, extracapsular cataract extraction (ECCE) or intracapsular cataract extraction (ICCE). IOLs used were either posterior chamber (PC IOL) or anterior chamber (AC IOL). Details of date of operation, type of surgery, IOL and intraoperative complications were recorded. Follow-up visits were on the first day, between 1–3 weeks and at 4–11 weeks. PVA and BCVA measured with a pin-hole at these follow-up visits were recorded. In order to assess outcome, visual acuities were categorized according to WHO guidelines on the outcome of cataract surgery: good (6/6-6/18), borderline (6/24-6/60), or poor surgical outcomes, (<6/60).

For statistical analysis, age was categorized into four categories: 40–49 years; 50–59 years; 60–69 years and ≥70 years of age. All comorbidities and intraoperative complications were categorized as present or absent. The secondary centers of the examination and operation were divided into three categories depending on the number of eyes operated in each center over the course of the study i.e those with less than 100 surgeries, 100–300 surgeries and those with >300 surgeries. Since there was only 1 ICCE case in the data, ICCE was excluded from statistical analysis.

For statistical analysis, Stata 11 was used [17]. Association of risk factors with visual outcomes was evaluated by logistic regression model and for categorical variable chi-squared or Fisher’s Exact test was used. A two tailed p value of <0.05 was considered statistically significant. Risk factors and poor outcomes were analyzed using univariable and multivariable regressions using data for BCVA at 4–11 weeks follow up. Good outcome was defined as a BCVA of 6/6-6/18. WHO categories of borderline and poor outcome (BCVA of <6/18-6/60 and <6/60) were used to define poor outcome. Multi-collinearity between variables was assessed by looking at the variance inflation factor and fitness of the model was assessed using Hosmer Lemeshow test for goodness of fit.

## Results

Between October 2009-March 2010, 3700 cataract surgeries were performed and 2049 age-related cataract surgeries records were randomly selected using a random number table. Table 1 shows the demographic and ocular characteristics of patients included in the study. The mean age was 61.8 years (SD: 8.9 years) and 1,133 (55.3%) surgeries were performed on female patients. Pre-existing ocular co-morbidity was present in 165 patients (8.1%).

**Table 1:** Baseline demographic and ocular characteristics.

Variables	N = 2049 (%)
**Age group (years)**	
40–49	169 (8.3)
50–59	376 (18.4)
60–69	1026 (50.1)
≥ 70	478 (23.3)
**Gender**	
Male	916 (44.8)
Female	1133 (55.3)
**Operated Eye**	
Right	1165 (56.9)
Left	884 (43.1)
Pre-operative BCVA*	
6/6-6/18	312 (15.2)
6/24-6/60	503 (24.6)
<6/60	1234 (60.2)
**Lens Status of Fellow Eye**	
Clear	26 (1.3)
Opacity	45 (2.2)
Operable Cataract	1492 (72.8)
Inoperable Cataract	19 (0.9)
Aphakia	6 (0.3)
Pseudophakia	457 (22.3)
Cannot Examine	4 (0.2)
**Preoperative Comorbidities in Eye Undergoing Surgery**	
Normal	1884 (92)
Corneal scar	34 (1.7)
Old iritis	6 (0.3)
Retinal disease	33 (1.6)
Glaucoma	18 (0.9)
Other	74 (3.6)

*BCVA = best corrected visual acuity

The most common procedure was SICS with PCIOL (91.8%) (Table 2). Intraoperative complication was seen in 29 eyes (1.4%) with the most common being striate keratopathy (13 eyes) followed by posterior capsular rent or zonular dehiscence (6 eyes).

**Table 2:** Type of cataract surgery performed at each of eight secondary hospitals.

Type of Surgery	1	2	3	4	5	6	7	8	Total
Phaco* + PC-IOL^$^	46	29	25	5	1	13	0	4	123
SICS^#^ + PC-IOL	617	354	193	118	90	363	31	116	1882
SICS + AC-IOL^@^	1	0	1	1	0	0	0	0	3
SICS + No IOL	1	0	2	1	0	0	1	0	5
ECCE^ + PC-IOL	0	2	0	18	1	2	10	0	33
ECCE + No IOL	1	0	0	0	0	1	0	0	2
ICCE + No IOL	1	0	0	0	0	0	0	0	1
Total	667	385	221	143	92	379	42	120	2049

*Phaco = phacoemulsification;

^#^SICS = small incision cataract surgery;

^ECCE = extracapsular cataract extraction;

^$^PC-IOL = posterior chamber intraocular lens;

^@^AC-IOL = anterior chamber intraocular lens

Table 3 shows the pre-operative visual acuity and outcome of cataract surgeries at different follow-up visit. One hundred and seventy eight patients (8.6%) missed the 1–3 week follow-up visit and 608 (29.7%) missed the 4–11 weeks follow-up visit. At 4–11 weeks follow-up visit, based on PVA, 61.8% had good outcome and based on BCVA, 91.7% had good outcome. Based on PVA and BCVA, those with less than 6/60 were only 2.9% and 1.6% respectively.

**Table 3:** Visual acuity at follow-up visits.

Visual acuity categories	Pre-operative visual acuity of operated eyes N = 2049 (100%)		Post-operative day 1 N = 2049 (100%)		Post-operative 1–3 weeks N = 1871 (91.3%)		Post-operative 4–11 weeks N = 1441 (70.3%)	
	PVA**	BCVA*	PVA**	BCVA*	PVA	BCVA	PVA	BCVA
6/18 or better	76 (3.7)	312 (15.2)	1069 (52.2)	1525 (74.4)	1217 (65.1)	1647 (88)	891 (61.8)	1322 (91.7)
<6/18-6/60	480 (23.4)	503 (24.6)	732 (35.7)	344 (16.8)	566 (30.3)	166 (8.9)	508 (35.3)	96 (6.7)
<6/60	1493 (72.9)	1234 (60.2)	248 (12.1)	180 (8.8)	88 (4.7)	58 (3.1)	42 (2.9)	23 (1.6)

**PVA = Presenting visual acuity;

*BCVA = best corrected visual acuity

Those lost to follow-up were significantly older, had fewer ocular co-morbidities and had ECCE as a procedure performed (Table 4).

**Table 4:** Demographic and ocular characteristics of those followed-up versus not followed-up at 4–11 Week Follow Up.

Variable	Available (%)	Not available (%)	P value
	N = 1441	N = 608	
**Age group (years)**			
40–49	127 (8.8)	42 (6.9)	
50–59	285 (19.8)	91 (15)	
60–69	739 (51.3)	287 (47.2)	
≥70	290 (20.1)	188 (30.9)	
			p<0.001
**Gender**			
Male	626 (43.4)	290 (47.7)	
Female	815 (56.6)	318 (52.3)	
			p = 0.07
**Secondary Hospital**			
<100	88 (6.1)	46 (7.6)	
100–300	354 (24.6)	130 (21.4)	
>300	999 (69.3)	432 (71.1)	
			p = 0.18
**Pre-operative Comorbidities**			
No	1314 (91.2)	570 (93.8)	
Yes	127 (8.8)	38 (6.3)	
			P = 0.05
**Intra-operative Complications**			
No	1423 (99)	597 (98.8)	
Yes	15 (1)	7 (1.2)	
			p = 0.82
**Intraocular lens**			
PC-IOL^$^	1432 (99.4)	606 (99.7)	
No IOL or AC-IOL^@^	9 (0.6)	2 (0.3)	
			p = 0.4
**Type of Surgery**			
Phacoemulsification	94 (6.5)	29 (4.8)	
SICS ^#^	1340 (93.1)	550 (90.5)	
ECCE^	6 (0.4)	29 (4.8)	
			p<0.001

^#^SICS = small incision cataract surgery;

^ECCE = extracapsular cataract extraction;

^$^PC-IOL = posterior chamber intraocular lens;

^@^AC-IOL = anterior chamber intraocular lens

As there were no significant differences of visual outcome at discharge, 1–3 weeks follow up, and 4–11 weeks follow up based on covariates, such as age, gender, ocular comorbidities, intraoperative complications, and type of surgery (data not shown), we used visual outcome at the 4–11 weeks follow up for further statistical analysis. Table 5 shows the demographic and ocular characteristics of those with good outcome (BCVA 6/6-6/18) versus poor outcome (BCVA <6/18). Increasing age, preoperative comorbidities, intraoperative complications, those undergoing ECCE, and those without IOL or with AC IOL had significantly poor visual outcome (X^2^ test, p<0.05).

**Table 5:** Demographic and ocular characteristics of those with good outcome (BCVA 6/6-6/18) versus poor outcome (BCVA <6/18).

Variable	6/6-6/18	<6/18	P value
**Age group (years)**			
40–49	122 (9.2)	5 (4.2)	
50–59	267 (20.2)	18 (15.1)	
60–69	687 (52.0)	52 (43.7)	
≥70	246 (18.6)	44 (37.0)	
			p<0.001
**Gender**			
Male	582 (44.0)	44 (36.9)	
Female	740 (56)	75 (63.0)	
			p = 0.14
**Secondary Hospital**			
<100	76 (86.4)	12 (13.6)	
100–300	322 (91.0)	32 (9.0)	
>300	924 (92.5)	75 (7.5)	
			p = 0.11
**Preoperative Comorbidities**			
No	1228 (92.9)	86 (72.3)	
Yes	94 (7.1)	33 (27.7)	
			p<0.001
**Intraoperative Complications**			
No	1311 (99.4)	112 (94.1)	
Yes	11 (0.8)	7 (5.9)	
			p<0.001
**Intraocular lens**			
PC-IOL^$^	1318 (99.7)	114 (95.8)	
No IOL or AC-IOL^@^	4 (0.3)	5 (4.2)	
			p<0.001
**Type of Surgery**			
Phacoemulsification	89 (6.7)	5 (4.2)	
SICS ^#^	1229 (93.0)	111 (93.3)	
ECCE^	3 (0.2)	3 (2.5)	
			p<0.001

^#^SICS = small incision cataract surgery;

^ECCE = extracapsular cataract extraction;

^$^PC-IOL = posterior chamber intraocular lens;

^@^AC-IOL = anterior chamber intraocular lens

To further analyze the associations between the potential predictors listed above and visual outcomes, univariable and multivariable logistic regressions were conducted (Table 6). Poor visual outcomes were significantly higher in patients aged ≥70 (OR 4.63; 95% CI 1.61, 13.30), in females (OR 1.58; 95% CI 1.04, 2.41), those with preoperative comorbidities (odds ratio 4.68; 95% CI 2.90, 7.57), with intraoperative complications (OR 8.01; 95% CI 2.91, 22.04), eyes that underwent no IOL or AC-IOL (OR 12.63; 95% CI 2.65, 60.25) and those undergoing ECCE (OR 9.39; 95% CI 1.18, 74.78). These associations were consistent even when age was used as continuous variable as well as with the forward and backward stepwise regressions.

**Table 6:** Univariable and multivariable association of demographic, location and surgical factors with visual outcome at 4–11 weeks follow-up.

Variable	Odds Ratio (95% CI)	Odds Ratio (95% CI)
	Univariable	Multivariable
**Age group (years)**		
40–49	Reference	Reference
50–59	1.64 (0.60, 4.53)	2.11 (0.70, 6.37)
60–69	1.85 (0.72, 4.72)	2.19 (0.78, 6.17)
≥70	4.36 (1.69, 11.29)	4.63 (1.61, 13.30)
Overall	p = <0.001	p = 0.001
**Gender**		
Male	Reference	Reference
Female	1.34 (0.91, 1.98)	1.58 (1.04, 2.41)
**Secondary Hospital**		
<100	Reference	Reference
100–300	0.63 (0.31, 1.28)	0.72 (0.34, 1.56)
>300	0.51 (0.27, 0.99)	0.56 (0.28, 1.15)
Overall	p = 0.12	P = 0.22
**Preoperative Comorbidities**		
No	Reference	Reference
Yes	5.01 (3.19, 7.88)	4.68 (2.90, 7.57)
**Intraoperative Complications**		
No	Reference	Reference
Yes	7.45 (2.83, 19.59)	8.01 (2.91, 22.04)
**Intraocular lens**		
PC-IOL^$^	Reference	Reference
No IOL or AC-IOL^@^	14.45 (3.83, 54.57)	12.63 (2.65, 60.25)
**Type of Surgery**		
Phacoemulsification	Reference	Reference
SICS ^#^	1.61 (0.64, 4.04)	1.24 (0.48, 3.19)
ECCE^	17.81 (2.84, 111.68)	9.39 (1.18, 74.78)
Overall	p = 0.008	p = 0.09
Goodness of Fit		0.24

^#^SICS = small incision cataract surgery;

^ECCE = extracapsular cataract extraction;

^$^PC-IOL = posterior chamber intraocular lens;

^@^AC-IOL = anterior chamber intraocular lens

## Discussion

This retrospective study focused on cataract surgery outcomes in 8 secondary care centers of LVPEI. Visual acuity tends to improve till 1–3 weeks follow-up visit. Subsequently, there was no difference between 1–3 weeks visit and 4–11 weeks follow-up At 4–11 weeks follow-up, based on PVA, there were 61.8% who had good outcome, with best-corrected, 91.7% had good outcomes. Though the PVA was less than that recommended by WHO (80%), the BCVA was well within the WHO recommendation, i.e. 90% BCVA having good outcome and <5% having less than 6/60 [18]. The outcomes are similar to those reported by some [4–7,19] and better than others from developing countries [20–24]. However, outcomes based on PVA was less than those described by Desai et.al from United Kingdom (UK) as most of the surgeries in the UK were phacoemulsification. [25] As most of the surgeries were MSICS in this study, it is likely that there would a post-operative refractive error correction would be needed in contrast with the case in UK. This signifies the importance of post-operative refraction and spectacles for cases operated by MSICS.

As seen in other studies, increasing age was one of the risk factor for poor outcome [4,7,26–28] Despite controlling for ocular comorbidities, increasing age was found to be one of the predictor for poor outcome. It is likely that those older had a denser cataract and therefore ocular co-morbidities in this group of patients were missed pre-operatively.

Female gender was also a risk factor for poor outcome. [26] Others have reported this risk factor in various studies [28]. Contradictory to our report, Gogate et.al and Venkatesh et.al, found good outcome in female patients. [4,5] It is likely that females were assessed for cataract at a later stages than male and had worse cataract, thus obscuring the pre-existing ocular comorbidity/condition, which is likely to affect outcomes.

Similar to other studies, preoperative comorbidities, [4,7,28] intraoperative complications, [4,7,24,26,27] and eyes with no IOL or AC-IOL were also risk factor for poor outcome. [4].

Those undergoing ECCE were also risk factor for poor outcome. It is likely that MSICS was the procedure of choice and ECCE was only performed in difficult cases where the surgeon deemed that MSICS would not be possible. This includes cases with small pupil, pseudo exfoliation, hazy cornea etc. Gogate et.al also found that, over a period of time, as they shifted from ECCE to MSICS, there was an improvement in outcomes. [4]

### Intraoperative Complications

Intraoperative complications were acquired in 29 (1.4%) of eyes in our data, which is similar to some studies done in China [26], Africa [21] and India [5] and much less than other studies reported from Hong Kong [24], China [27,29] and Nigeria [23]. The difference is due to skill level of the surgeon operating, inclusion criteria etc. For example, in Hong Kong, all those operated were 90 years and above, thus had higher associated ocular co-morbidities. Similarly, in China, novice surgeons performed surgeries [29]. Thus proper pre-operative evaluation, uniform and proper training as well as providing adequate infrastructure and equipment support may help in reduction of post-operative complications.

### Ocular Comorbidities

In our study, preoperative, ocular comorbidities were present in 8.1% of eyes undergoing surgery. This was less than some studies from India [4] and China [24,29] as well as some developed countries [30] but more than other studies from China [26] and similar to a study from Africa [7] and Iran [28]. The difference is mainly due to the difference in age group included in the studies, pre-operative evaluation and density of cataract obscuring posterior segment pathologies.

### Limitations

Nearly 10% of the total patient did not return for 1–3 weeks follow-up and a third did not return for the 4–11 week follow up. This is similar to follow up rates seen in other developing countries [6,23]. It is likely that there were issues related to accessing services for those who did not return for follow-up and this needs to be further investigated. However, in terms of outcomes, there was no difference in these groups. Strategies to improve follow-up including reimbursement for transport, free spectacles, sending reminders, adequate counseling etc could also be considered to improve follow-up. Another limitation was that individual surgeon level data was not collected and analyzed as we assumed that all surgeons in this study were adequately trained using a uniform protocol for 18 months at L V Prasad Eye Institute, certified before they were posted in rural secondary centres and provided good surgical facility and equipment (operating microscope, A-scan etc) too. Hence we assumed that there would be minimal difference between these surgeons.

## Conclusion

Our study indicates that quality cataract surgeries can be achieved at any size of rural secondary centers. In addition, adequate and uniform training protocol, following uniform systems at all levels of care, provision of good surgical facility and equipment (operating microscope, A-scan etc) and regular monitoring could improve the outcomes in any rural setting. Apart from that adequate and long-term management of complications would be good way to improve outcomes.

At the same time, monitoring outcomes of cataract surgery should be done routinely in all hospitals so that trends can be monitored over time. Studies have also shown that, if monitored, cataract surgery outcomes tend to improve over time. [4,7] Monitoring should not be used as a tool to compare individual surgeons / centres and those with ocular co-morbidities should not be denied cataract surgeries.
